# Supplementary material for: Implications of tree expansion in shrubland ecosystems for two generalist avian predators
Source: PLoS One. 2023 Jun 2;18(6):e0286478. doi: 10.1371/journal.pone.0286478 (PMC10237380; doi:10.1371/journal.pone.0286478)
Supplement: S1 File — Model rankings for the the effects of habitat structure and prey covariates on habitat use and the effect of habitat characteristics on detection of common raven (Corvus corax) and red-tailed hawk (Buteo jamaicensis) in southwest Idaho, 2017–2020. (DOCX) [file pone.0286478.s001.docx]

Table A1. Model rankings for the effect of juniper (*Juniperus occidentalis*) on habitat use by common ravens (*Corvus corax*) in the Owyhee Mountains of southwestern Idaho, 2017-2020. We conducted 409 occupancy surveys at 37 transects. We tested nine spatial scales of percent juniper cover within 100 m to 3000 m. We tested quadratic models for each spatial scale. We also tested for an effect of the proportion of phases one (<10% juniper cover), two (10-20% juniper cover), and three (>20% juniper cover) at each spatial scale. Finally, we tested for an effect of tree clustering on raven occupancy using a nearest neighbor algorithm value generated using a 1 m resolution juniper layer at each spatial scale. We used Bayesian multi-season models and compared models using leave-one-out cross validation.

| **Model** | **^1^elpd_diff** | **^2^se_diff** | **^3^*p* value** |
| --- | --- | --- | --- |
| ^4^100 p3 | 0.00 | 0.00 | 0.35 |
| 250 p3 | -1.41 | 5.43 | 0.31 |
| 100 | -2.85 | 5.86 | 0.36 |
| 250 | -4.53 | 5.89 | 0.32 |
| 750*750 | -4.93 | 1.21 | 0.19 |
| 1000*1000 | -5.53 | 1.92 | 0.19 |
| 100*100 | -6.23 | 4.17 | 0.26 |
| 250*250 | -7.18 | 3.59 | 0.24 |
| 500 p3 | -7.89 | 5.42 | 0.21 |
| 100 p2 | -8.11 | 5.50 | 0.24 |
| 500*500 | -8.25 | 2.51 | 0.21 |
| 500 | -9.65 | 5.90 | 0.23 |
| 250 p2 | -10.34 | 5.52 | 0.20 |
| 750 p3 | -11.51 | 5.43 | 0.17 |
| 750 | -12.83 | 5.92 | 0.18 |
| ^5^500 cl | -12.94 | 4.88 | 0.19 |
| 750 cl | -13.41 | 4.83 | 0.18 |
| 3000 p2 | -13.43 | 7.03 | 0.09 |
| 2000^2^ | -13.49 | 3.96 | 0.13 |
| 3000 cl | -13.72 | 4.66 | 0.17 |
| 1000 cl | -13.90 | 4.79 | 0.16 |
| 1000 p3 | -13.94 | 5.52 | 0.14 |
| 2500 cl | -14.06 | 4.75 | 0.16 |
| 2500 p3 | -14.12 | 5.71 | 0.12 |
| 1500 cl | -14.16 | 4.79 | 0.15 |
| 2000 p3 | -14.19 | 5.73 | 0.12 |
| 2000 | -14.21 | 6.15 | 0.14 |
| 2000 cl | -14.22 | 4.79 | 0.15 |
| 1500 p3 | -14.26 | 5.61 | 0.12 |
| 1000 | -14.32 | 5.96 | 0.16 |
| 500 p2 | -14.32 | 5.85 | 0.13 |
| 3000 p1 | -14.48 | 7.00 | 0.08 |
| 1500 | -14.56 | 6.04 | 0.15 |
| 2500 | -14.68 | 6.21 | 0.13 |
| 2500 p2 | -14.70 | 6.72 | 0.08 |
| 3000 | -14.80 | 6.28 | 0.12 |
| 3000 p3 | -14.80 | 5.80 | 0.11 |
| 250 cl | -14.85 | 4.71 | 0.14 |
| 2500^2^ | -14.92 | 4.36 | 0.12 |
| 100 cl | -15.10 | 4.93 | 0.16 |
| 2000 p2 | -15.22 | 6.54 | 0.09 |
| 2500 p1 | -15.28 | 6.79 | 0.08 |
| 100 p1 | -15.35 | 4.97 | 0.15 |
| 250 p1 | -15.63 | 5.07 | 0.14 |
| 1000 p2 | -15.84 | 5.95 | 0.10 |
| 750 p2 | -15.90 | 5.95 | 0.10 |
| 2000 p1 | -16.04 | 6.44 | 0.09 |
| 1500 p2 | -16.10 | 6.09 | 0.09 |
| 750 p1 | -16.70 | 5.58 | 0.11 |
| 500 p1 | -16.70 | 5.65 | 0.11 |
| 1500 p1 | -16.72 | 6.07 | 0.09 |
| 1000 p1 | -16.73 | 5.76 | 0.10 |
| 3000*3000 | -16.90 | 4.95 | 0.06 |
| ^1^ Expected log predictive density. Larger scores indicate the model is more predictive.  ^2^ Standard error of the difference in elpd between a model and the most predictive model.  ^3^ Bayesian p value calculated using the Freeman-Tukey test statistic. Values closer to 0.5 indicate a better model fit, ^4^ Phase 1, Phase 2, Phase 3  ^5^ Tree Clustering | | | |

Table A2. Habitat model rankings for common raven (*Corvus corax*) habitat use in southwest Idaho, 2017-2020. We conducted 409 occupancy surveys at 37 transects. We used Bayesian multi-season models to test the effects of habitat variables on common raven occupancy and compared models using leave-one-out cross validation.

| **Model** | **^1^elpd_diff** | **^2^se_diff** | **^3^*p-*value** |
| --- | --- | --- | --- |
| Juniper cover + water | 0.00 | 0.00 | 0.35 |
| Juniper cover | -0.80 | 1.48 | 0.31 |
| Juniper cover + road | -4.30 | 1.38 | 0.29 |
| Null | -6.83 | 2.00 | 0.23 |
| Juniper cover + cabin | -7.05 | 1.94 | 0.29 |
| Water | -7.40 | 1.59 | 0.21 |
| Cliff + stream | -9.90 | 6.02 | 0.09 |
| Juniper + cliff | -10.93 | 5.22 | 0.11 |
| Cliff | -11.01 | 5.57 | 0.09 |
| Stream | -11.74 | 2.46 | 0.14 |
| Road | -12.17 | 2.52 | 0.14 |
| Cabin | -12.52 | 2.81 | 0.15 |
| Juniper removal | -14.67 | 3.07 | 0.13 |
| ^1^ Expected log predictive density. Larger scores indicate the model is more predictive.  ^2^ Standard error of the difference in elpd between a model and the most predictive model.  ^3^ Bayesian p-value calculated using the Freeman-Tukey test statistic. Values closer to 0.5 indicate a better model fit. | | | |

Table A3. Model rankings for common raven (*Corvus corax*) detection in southwest Idaho, 2017-2020. We conducted 409 occupancy surveys at 37 transects. We used Bayesian multi-season models to test the effects of habitat variables on common raven detection and compared models using leave-one-out cross validation.

| **Model** | **^1^elpd_diff** | **^2^se_diff** | **Bayesian *p-*value** |
| --- | --- | --- | --- |
| Terrain Roughness Index | 0.00 | 0.00 | 0.22 |
| Null | -1.30 | 2.70 | 0.19 |
| Time of Year | -2.70 | 2.80 | 0.19 |
| ^1^ Expected log predictive density. Larger scores indicate the model is more predictive.  ^2^ Standard error of the difference in elpd between a model and the most predictive model. | | | |

Table A4. Habitat model rankings for red-tailed hawk (*Buteo jamaicensis*) habitat use in southwest Idaho, 2017-2020. We conducted 409 occupancy surveys at 37 transects. We used Bayesian multi-season models to test the effects of habitat variables on red-tailed hawk occupancy and compared models using leave-one-out cross validation.

| **Model** | **^1^elpd_diff** | **^2^se_diff** | **^3^*p* value** |
| --- | --- | --- | --- |
| Cliff | 0.00 | 0.00 | 0.07 |
| Cliff + Tree Clustering | -0.26 | 1.36 | 0.07 |
| Water + Tree Clustering | -0.69 | 5.29 | 0.10 |
| Null | -1.45 | 3.97 | 0.15 |
| Water | -1.55 | 4.42 | 0.11 |
| Tree Clustering | -2.02 | 4.91 | 0.12 |
| Treatment | -2.80 | 3.82 | 0.10 |
| Cabins + Tree Clustering | -3.69 | 4.29 | 0.07 |
| Distance to Road | -4.08 | 4.02 | 0.06 |
| Distance to Cabins | -4.28 | 3.83 | 0.06 |
| Distance to Streams | -5.10 | 3.93 | 0.06 |
| Distance to Road + Tree Clustering | -5.39 | 4.88 | 0.05 |
| Distance to Stream + Tree Clustering | -7.17 | 4.68 | 0.04 |
| ^1^ Expected log predictive density. Larger scores indicate the model is more predictive.  ^2^ Standard error of the difference in elpd between a model and the most predictive model.  ^3^ Bayesian p-value calculated using the Freeman-Tukey test statistic. Values closer to 0.5 indicate a better model fit. | | | |

Table A5. Model rankings for the effect of juniper (*Juniperus occidentalis*) on habitat use by red-tailed hawks (*Buteo jamacensis*) in the Owyhee Mountains of southwestern Idaho, 2017-2020. We conducted 409 occupancy surveys at 37 transects. We tested nine spatial scales of percent juniper cover within 100 m to 3000 m. We tested quadratic models for each spatial scale. We also tested for an effect of the proportion of phases one (<10% juniper cover), two (10-20% juniper cover), and three (>20% juniper cover) at each spatial scale. Finally, we tested for an effect of tree clustering on occupancy using a nearest neighbor algorithm value generated using a 1-m resolution juniper layer at each spatial scale. We used Bayesian multi-season models and compared models using leave-one-out cross validation.

| **Model** | **^1^elpd_diff** | **^2^se_diff** | **^3^*p* value** |
| --- | --- | --- | --- |
| ^4^500 cl | 0.00 | 0.00 | 0.12 |
| 750 cl | -0.50 | 3.55 | 0.12 |
| 500*500 | -0.60 | 0.87 | 0.06 |
| 250 cl | -1.53 | 3.95 | 0.09 |
| 3000 cl | -2.13 | 3.75 | 0.10 |
| 3000*3000 | -2.22 | 2.39 | 0.06 |
| 100 cl | -2.46 | 3.16 | 0.11 |
| ^5^500 p2 | -2.59 | 2.72 | 0.08 |
| 1000 cl | -2.98 | 3.15 | 0.10 |
| 250 p2 | -3.45 | 2.75 | 0.07 |
| 2500 p1 | -3.51 | 3.87 | 0.08 |
| 1500*1500 | -3.51 | 2.46 | 0.04 |
| 3000 p1 | -3.53 | 4.17 | 0.07 |
| 750 p2 | -3.57 | 2.76 | 0.07 |
| 1500 cl | -3.59 | 3.54 | 0.08 |
| 2000 cl | -3.62 | 3.63 | 0.08 |
| 3000 | -3.63 | 3.13 | 0.07 |
| 500 p3 | -3.68 | 3.25 | 0.09 |
| 100 p3 | -3.79 | 3.19 | 0.09 |
| 750 p3 | -3.80 | 3.33 | 0.08 |
| 250*250 | -3.82 | 2.16 | 0.05 |
| 2500 cl | -3.84 | 3.61 | 0.08 |
| 750 | -3.84 | 3.23 | 0.08 |
| 1000 p3 | -3.90 | 3.32 | 0.08 |
| 2000 p1 | -3.91 | 3.64 | 0.08 |
| 500 | -3.92 | 3.22 | 0.08 |
| 100 | -3.92 | 3.21 | 0.08 |
| 250 | -3.99 | 3.24 | 0.08 |
| 1000 p2 | -3.99 | 2.88 | 0.07 |
| 1500 p1 | -4.07 | 3.52 | 0.08 |
| 1500 | -4.08 | 3.21 | 0.08 |
| 100 p2 | -4.08 | 3.13 | 0.08 |
| 1000 | -4.11 | 3.24 | 0.08 |
| 1500 p3 | -4.12 | 3.27 | 0.08 |
| 2000 | -4.13 | 3.18 | 0.08 |
| 2000 p2 | -4.16 | 3.25 | 0.08 |
| 2000 p3 | -4.17 | 3.30 | 0.08 |
| 250 p1 | -4.19 | 2.64 | 0.06 |
| 1000*1000 | -4.22 | 2.33 | 0.05 |
| 750 p1 | -4.25 | 3.03 | 0.07 |
| 100 p1 | -4.26 | 3.08 | 0.07 |
| 3000 p2 | -4.26 | 3.38 | 0.08 |
| 3000 | -4.30 | 3.18 | 0.08 |
| 2500 p3 | -4.36 | 3.27 | 0.08 |
| 250 p3 | -4.37 | 3.25 | 0.08 |
| 1000 p1 | -4.42 | 3.14 | 0.07 |
| 2500*2500 | -4.45 | 2.43 | 0.05 |
| 500 p1 | -4.49 | 3.02 | 0.07 |
| 2500 p2 | -4.50 | 3.36 | 0.07 |
| 1500 p2 | -4.55 | 3.23 | 0.07 |
| 2000*2000 | -4.58 | 2.39 | 0.05 |
| 3000 p3 | -4.65 | 3.28 | 0.07 |
| 750*750 | -5.15 | 2.32 | 0.04 |
| 100*100 | -5.79 | 2.71 | 0.04 |
| ^1^ Expected log predictive density. Larger scores indicate the model is more predictive.  ^2^ Standard error of the difference in elpd between a model and the most predictive model.  ^3^ Bayesian p value calculated using the Freeman-Tukey test statistic. Values closer to 0.5 indicate a better model fit.  ^4^ Phase 1, Phase 2, Phase 3  ^5^ Tree Clustering | | | |

Table A6. Model rankings for red-tailed hawk (*Buteo jamaicensis*) detection in southwest Idaho, 2017-2020. We conducted 409 occupancy surveys at 37 transects. We used Bayesian multi-season models to test the effects of habitat variables on red-tailed hawk detection and compared models using leave-one-out cross validation.

| **Model** | **^1^elpd_diff** | **^2^se_diff** |
| --- | --- | --- |
| Null | 0.00 | 0.00 |
| Time of Year | -0.50 | 0.90 |
| ^3^Terrain Roughness Index | -1.3 | 0.60 |
| ^1^ Expected log predictive density. Larger scores indicate the model is more predictive.  ^2^ Standard error of the difference in elpd between a model and the most predictive model. | | |

Table A7. Prey model rankings for common raven (*Corvus corax*) occupancy in southwest Idaho 2017-2020. We conducted 409 occupancy surveys at 37 transects. We used Bayesian multi-season models to test the effects of habitat variables on common raven occupancy and compared models using leave-one-out cross validation.

| **Model** | **^1^elpd_diff** | **^2^se_diff** | **^3^*p* value** |
| --- | --- | --- | --- |
| ^4^Songbirds + ^5^Juniper | 0 | 0 | 0.35 |
| Juniper | -1.62 | 1.84 | 0.31 |
| ^6^Small Mammals + Juniper | -4.89 | 2.15 | 0.30 |
| Songbirds*Juniper | -5.00 | 0.69 | 0.30 |
| Small Mammals*Juniper | -5.26 | 2.22 | 0.29 |
| ^7^Ground Squirrels + Juniper | -5.30 | 3.20 | 0.24 |
| Small Mammals | -13.55 | 2.50 | 0.16 |
| Songbirds | -13.93 | 2.65 | 0.16 |
| Ground Squirrels | -14.40 | 3.22 | 0.14 |
| ^8^All prey groups additive | -20.70 | 3.60 | 0.05 |
| ^1^ Expected log predictive density. Larger scores indicate the model is more predictive.  ^2^ Standard error of the difference in elpd between a model and the most predictive model.  ^3^ Bayesian p-value calculated using the Freeman-Tukey test statistic. Values closer to 0.5 indicate a better model fit, ^4^ Unadjusted count, ^5^ % *Juniperus occidentalis* cover 100 m, ^6^ Density/ha, ^7^ Presence/absence, ^8^ Songbirds + small mammals + ground squirrels. | | | |
|  | | | |

Table A8. Prey model rankings for red-tailed hawk (*Buteo jamaicensis*) occupancy in southwest Idaho, 2017-2020. We conducted 409 occupancy surveys at 37 transects. We used Bayesian multi-season models to test the effects of habitat variables on red-tailed hawk occupancy and compared models using leave-one-out cross validation.

| **Model** | **^1^elpd_diff** | **^2^se_diff** | **^3^*p* value** |
| --- | --- | --- | --- |
| ^4^Songbird + ^5^Cliff | 0.00 | 0.00 | 0.08 |
| Songbird*Cliff | -1.85 | 1.16 | 0.08 |
| ^6^Small mammals*Cliff | -4.67 | 3.36 | 0.09 |
| Cliff | -4.79 | 2.18 | 0.07 |
| ^7^All prey groups additive | -5.59 | 5.32 | 0.06 |
| Small mammals + Cliff | -5.59 | 2.46 | 0.06 |
| ^8^Ground squirrels + Cliff | -6.43 | 2.03 | 0.06 |
| Songbirds | -8.54 | 4.89 | 0.08 |
| Small mammals | -9.67 | 5.54 | 0.08 |
| Ground squirrels | -9.92 | 5.18 | 0.07 |
| ^1^ Expected log predictive density. Larger scores indicate the model is more predictive.  ^2^ Standard error of the difference in elpd from the most predictive model.  ^3^ Bayesian p value calculated using the Freeman-Tukey test statistic. Values closer to 0.5 indicate a better model fit.  ^4^ Unadjusted count, ^5^ Distance m, ^6^ Density/ha, ^7^Songbirds + small mammals + ground squirrels, ^8^ Presence/absence. | | | |
|  | | | |
