# Supplementary material for: Implications of tree expansion in shrubland ecosystems for two generalist avian predators
Source: PLoS One. 2023 Jun 2;18(6):e0286478. doi: 10.1371/journal.pone.0286478 (PMC10237380; doi:10.1371/journal.pone.0286478)
Supplement: S1 Data — (ZIP) [file pone.0286478.s002.zip › S1_File/~WRL1892.tmp]

Table 2.1. Covariates used to test effects of habitat features on detection and occupancy probability of common ravens (*Corvus corax*) and red-tailed hawks (*Buteo jamaicensis)* in southwest Idaho, 2017-2020. We used a three-stage modeling approach, carrying over the top ranked model from each stage. We ranked models using leave one out cross validation and Bayesian p-values.

| **Detection** | **Habitat Structure Models** | **Prey Models** |
| --- | --- | --- |
| Null | Null | Structure model (sm) |
| Time of year | Juniper Cover | Small mammals |
| Terrain roughness index | Distance to cliff | Songbirds |
|  | Distance to water | Ground squirrels |
|  | Distance to stream | Small mammals + sm |
|  | Distance to road | Songbirds + sm |
|  | Distance to human dwelling | Ground squirrels + sm |
|  | Distance to cliff + juniper | Small mammals*sm |
|  | Distance to cliff + distance to stream | Songbirds*sm |
|  | Distance to road + juniper | All prey groups additive |
|  | Distance to water + juniper |  |
|  | Distance to human dwelling + juniper |  |
|  | Small mammal density |  |
|  | Juniper Removal |  |
|  |  |  |

Table A5. Prey model rankings for common raven (*Corvus corax*) occupancy in southwest Idaho 2017-2020. We conducted 409 occupancy surveys at 37 transects. We used Bayesian multi-season models to test the effects of habitat variables on common raven occupancy and compared models using leave-one-out cross validation.

| **Model** | **^1^elpd_diff** | **^2^se_diff** | **^3^*p* value** |
| --- | --- | --- | --- |
| ^4^Songbirds + ^5^Juniper | 0 | 0 | 0.35 |
| Juniper | -1.62 | 1.84 | 0.31 |
| ^6^Small Mammals + Juniper | -4.89 | 2.15 | 0.30 |
| Songbirds*Juniper | -5.00 | 0.69 | 0.30 |
| Small Mammals*Juniper | -5.26 | 2.22 | 0.29 |
| ^7^Ground Squirrels + Juniper | -5.30 | 3.20 | 0.24 |
| Small Mammals | -13.55 | 2.50 | 0.16 |
| Songbirds | -13.93 | 2.65 | 0.16 |
| Ground Squirrels | -14.40 | 3.22 | 0.14 |
| ^8^All prey groups additive | -20.70 | 3.60 | 0.05 |
| ^1^ Expected log predictive density. Larger scores indicate the model is more predictive.  ^2^ Standard error of the difference in elpd between a model and the most predictive model.  ^3^ Bayesian p-value calculated using the Freeman-Tukey test statistic. Values closer to 0.5 indicate a better model fit, ^4^ Unadjusted count, ^5^ % *Juniperus occidentalis* cover 100 m, ^6^ Density/ha, ^7^ Presence/absence, ^8^ Songbirds + small mammals + ground squirrels | | | |
|  | | | |
