# Supplementary material for: Implications of tree expansion in shrubland ecosystems for two generalist avian predators
Source: PLoS One. 2023 Jun 2;18(6):e0286478. doi: 10.1371/journal.pone.0286478 (PMC10237380; doi:10.1371/journal.pone.0286478)
Supplement: S1 Data — (ZIP) [file pone.0286478.s002.zip › S1_File/Read Me Young et al 2023.docx]

**Abstract:**

Shrublands globally have undergone structural changes due to plant invasions, including the expansion of native trees. Removal of native conifer trees, especially juniper (*Juniperus spp.*), is occurring across the Great Basin of the western U.S. to support declining sagebrush (*Artemisia* spp.) habitats and associated wildlife species, such as greater sage-grouse (*Centrocercus urophasianus*). One justification for conifer removal is that it may improve survival of sagebrush-associated wildlife by reducing the abundance of avian predators. However, the relationship between conifer expansion and predator distributions has not been explicitly evaluated. Further, although structural characteristics of habitat are important for generalist predators, overall prey abundance may also affect habitat use by predators. We examined habitat use of common ravens (*Corvus corax*) and red-tailed hawks (*Buteo jamaicensis*), two generalist predators whose populations are increasing in western North America, to variation in structural characteristics and prey distributions in sagebrush habitat that has experienced conifer expansion. Structural characteristics of habitat were important predictors of habitat use for both ravens and red-tailed hawks, whereas measures of prey abundance were unimportant for both species likely because generalist predators can use a wide variety of food resources. Ravens, but not red-tailed hawks, responded positively to increasing cover of juniper and the probability of habitat use was highest (> 0.95) where juniper cover within 100 m was > 20%. Habitat use by red-tailed hawks, but not ravens, was greater near cliffs but was not associated with juniper cover.

Our study suggests that the removal of conifer in similar environments may lower the probability of habitat use for ravens, a common predator with significant impacts on many prey species. Therefore, we suggest conifer removal may improve sage-grouse reproductive success and survival depending on responses to conifer removal from other predators. Our results may be reflective of similar changes in rangeland ecosystems around the world undergoing expansion of conifer and other woody vegetation. Though species identities differ from sagebrush habitats, generalist avian predators in other habitats may have similar relationships with structural resources.2017-2020 RR Juniper - % juniper cover within nine spatial scales around each survey transect.

**Data**:

PRPA rr mean density – predicted mean density of Great Basin pocket mice (*Perognathus parvus*, PRPA) for each survey transect. This variable was created using the most predictive vegetation variables for PRPA density. For this species proportion of sagebrush cover estimated using National Land Cover Database, 2016 spatial layers with a 30m^2^ resolution. We predicted PRPA density at all avian predator survey transects using the relationship between vegetation and small mammal density estimated at 14 sampling locations for small mammals. See Young 2022

PEMA rr mean density - predicted mean density of deer mice (*Peromyscus maniculatus*, PEMA) for each survey transect. This variable was created using the most predictive vegetation variables for PEMA density. For this species juniper cover within 100 m best predicted density, and PEMA density was highest at 10% juniper cover. We predicted PEMA density at all avian predator survey transects using the relationship between vegetation and small mammal density estimated at 14 sampling locations for small mammals. See Young 2022

Ground squirrels – categorical data for ground squirrel presence/absence at each survey location. Present = 1, Absent = 0. Ground squirrel presence/absence was estimated using visual and auditory observations during avian predator surveys.

Structure Covariates - .csv, covariates used in models testing the effect of structure on avian predator occupancy.

Cliff – Distance (m) to the nearest cliff.

Road – Distance (m) to the nearest dirt road.

Triangle – Distance (m) to the nearest human dwelling

Dist_pond – Distance (m) to the nearest manmade pond

Dist_stream – Distance (m) to the nearest stream

- Distance to Water – derived, distance to closest stream or pond.

Tri1k_mean – Terrain Roughness Index, mean within 1 km of each survey transect.

Treatment – Categorical, juniper removed from survey transect = 1, juniper not removed = 0

Shrub height and density – mean height (cm) and density (/m^2^) of big sagebrush (*Artemisia tridentata*) for each survey transect. Measured *in situ,* see Young 2022

Juniper cover – Estimated using a supervised and classification in ESRI ArcGIS and proofed *in situ* using visual confirmation.

CORA occupancy data – folder, occupancy data for three visits during each of four years for common raven, 2017-2020. Naming convention: y17-y20 = occupancy over three visits from 2017 to 2020.

RTHA occupancy data – folder, occupancy data for three visits during each of four years for red-tailed hawk, 2017-2020. Naming convention: y17Rav-y20Rav = occupancy over three visits each year from 2017 to 2020.

**Code**:

Data Prep and Model Run.R – Code to format vegetation and prey distribution data and run the CORA dynamic occupancy model testing for an effect of habitat structure on occupancy.

Final CORA Model Text - .txt file, code for raven JAGS dynamic occupancy model with juniper cover within 100 m and distance to water (m) as a covariate. Red-tailed hawk models and models that test for an effect of prey distributions on avian predator occupancy have the same structure with various predictor covariates.
