# Supplementary material for: Implications of tree expansion in shrubland ecosystems for two generalist avian predators
Source: PLoS One. 2023 Jun 2;18(6):e0286478. doi: 10.1371/journal.pone.0286478 (PMC10237380; doi:10.1371/journal.pone.0286478)
Supplement: Attachment — Submitted filename: Response to Reviewers.docx [file pone.0286478.s004.docx]

PONE-D-22-29377R1

Implications of tree expansion in shrubland ecosystems on two generalist avian predators .

PLOS ONE

Dear Dr. Young,

Thank you for submitting your manuscript to PLOS ONE. After careful consideration, we feel that it has merit but does not fully meet PLOS ONE’s publication criteria as it currently stands. Therefore, we invite you to submit a revised version of the manuscript that addresses the points raised during the review process.

We are sorry to be late with your MS decision, but we were unable to contact one of the previous reviewers, so  we had to invite a new reviewer.

Despite this, both reviewers are very positive and have only shown minor concerns that are easily addressed by you.

Please submit your revised manuscript by Jun 12 2023 11:59PM. If you will need more time than this to complete your revisions, please reply to this message or contact the journal office at [plosone@plos.org](mailto:plosone@plos.org). When you're ready to submit your revision, log on to [https://www.editorialmanager.com/pone/](https://urldefense.com/v3/__https:/www.editorialmanager.com/pone/__;!!JYXjzlvb!iLp3dbk4hWOvVv8rjAK9_MYyifNe-tzSHHYZWw0KGWMmBHhCZvFaorZsdLsA36EDITdELUVP2G7TUGJY$) and select the 'Submissions Needing Revision' folder to locate your manuscript file.

Please include the following items when submitting your revised manuscript:

- A rebuttal letter that responds to each point raised by the academic editor and reviewer(s). You should upload this letter as a separate file labeled 'Response to Reviewers'.
- A marked-up copy of your manuscript that highlights changes made to the original version. You should upload this as a separate file labeled 'Revised Manuscript with Track Changes'.
- An unmarked version of your revised paper without tracked changes. You should upload this as a separate file labeled 'Manuscript'.

If you would like to make changes to your financial disclosure, please include your updated statement in your cover letter. Guidelines for resubmitting your figure files are available below the reviewer comments at the end of this letter.

If applicable, we recommend that you deposit your laboratory protocols in protocols.io to enhance the reproducibility of your results. Protocols.io assigns your protocol its own identifier (DOI) so that it can be cited independently in the future. For instructions see: [https://journals.plos.org/plosone/s/submission-guidelines#loc-laboratory-protocols](https://urldefense.com/v3/__https:/journals.plos.org/plosone/s/submission-guidelines*loc-laboratory-protocols__;Iw!!JYXjzlvb!iLp3dbk4hWOvVv8rjAK9_MYyifNe-tzSHHYZWw0KGWMmBHhCZvFaorZsdLsA36EDITdELUVP2C_TBS98$). Additionally, PLOS ONE offers an option for publishing peer-reviewed Lab Protocol articles, which describe protocols hosted on protocols.io. Read more information on sharing protocols at [https://plos.org/protocols?utm_medium=editorial-email&utm_source=authorletters&utm_campaign=protocols](https://urldefense.com/v3/__https:/plos.org/protocols?utm_medium=editorial-email&utm_source=authorletters&utm_campaign=protocols__;!!JYXjzlvb!iLp3dbk4hWOvVv8rjAK9_MYyifNe-tzSHHYZWw0KGWMmBHhCZvFaorZsdLsA36EDITdELUVP2MD4KrAy$).

We look forward to receiving your revised manuscript.

Kind regards,

Juan Manuel Pérez-García, PhD
Academic Editor
PLOS ONE

Journal Requirements:

Please review your reference list to ensure that it is complete and correct. If you have cited papers that have been retracted, please include the rationale for doing so in the manuscript text, or remove these references and replace them with relevant current references. Any changes to the reference list should be mentioned in the rebuttal letter that accompanies your revised manuscript. If you need to cite a retracted article, indicate the article’s retracted status in the References list and also include a citation and full reference for the retraction notice.

[Note: HTML markup is below. Please do not edit.]

Reviewers' comments:

Reviewer's Responses to Questions

**Comments to the Author**

1. If the authors have adequately addressed your comments raised in a previous round of review and you feel that this manuscript is now acceptable for publication, you may indicate that here to bypass the “Comments to the Author” section, enter your conflict of interest statement in the “Confidential to Editor” section, and submit your "Accept" recommendation.

Reviewer #2: All comments have been addressed

Reviewer #3: (No Response)

2. Is the manuscript technically sound, and do the data support the conclusions?

The manuscript must describe a technically sound piece of scientific research with data that supports the conclusions. Experiments must have been conducted rigorously, with appropriate controls, replication, and sample sizes. The conclusions must be drawn appropriately based on the data presented.

Reviewer #2: Yes

Reviewer #3: Yes

3. Has the statistical analysis been performed appropriately and rigorously?

Reviewer #2: Yes

Reviewer #3: Yes

4. Have the authors made all data underlying the findings in their manuscript fully available?

The [PLOS Data policy](https://urldefense.com/v3/__http:/www.plosone.org/static/policies.action*sharing__;Iw!!JYXjzlvb!iLp3dbk4hWOvVv8rjAK9_MYyifNe-tzSHHYZWw0KGWMmBHhCZvFaorZsdLsA36EDITdELUVP2M8OrbvD$) requires authors to make all data underlying the findings described in their manuscript fully available without restriction, with rare exception (please refer to the Data Availability Statement in the manuscript PDF file). The data should be provided as part of the manuscript or its supporting information, or deposited to a public repository. For example, in addition to summary statistics, the data points behind means, medians and variance measures should be available. If there are restrictions on publicly sharing data—e.g. participant privacy or use of data from a third party—those must be specified.

Reviewer #2: No

Reviewer #3: Yes

5. Is the manuscript presented in an intelligible fashion and written in standard English?

PLOS ONE does not copyedit accepted manuscripts, so the language in submitted articles must be clear, correct, and unambiguous. Any typographical or grammatical errors should be corrected at revision, so please note any specific errors here.

Reviewer #2: Yes

Reviewer #3: Yes

6. Review Comments to the Author

Please use the space provided to explain your answers to the questions above. You may also include additional comments for the author, including concerns about dual publication, research ethics, or publication ethics. (Please upload your review as an attachment if it exceeds 20,000 characters)

Reviewer #2: The manuscript is clear and easy to foollow, it is now appropiate for publication. I have included only a couple of minor comments related to editing and to the replicability of this work in terms of its statistical analyses.
Line 170. Replace project by study area: "The climate of the study area is ...".

*Thank you, we made this change on line 172*

I suggest including a JAGS code in an appendix for the replicability of the work in other contexts. I believe that this information will add value to the manuscript, as it is free software that is being included in an increasing way of scientific articles and open source journals.

*Thank you, we have included JAGS model code in the data submitted to PLOS one during the resubmission process and this code will be available freely.*

Reviewer #3: This is a well-written paper that uses a robust dataset to evaluate the implications of tree expansion over two generalist predator presence in a sagebrush ecosystem. Clearly a lot of work went into data collection, which includes predator species and his prey across a sagebrush steppe. The study has strong potential to be useful for management and conservation, but in my opinion there are some issues that need to be resolved before this paper is published.
The authors discuss potential implications of their study for the management and conservation of Sage-grouse but the data and results support this indirectly. Although data on the abundance of the potential prey of two generalist predators were used, there are no data on the abundance or presence of the Sage-grouse. In the same way, the authors argue that the abundance of prey does not influence the habitat use by the two species of predators, but only a part of the potential prey was studied. I suggest caution with the conclusions drawn from the results. Is it possible to predators are responding to another prey species present in the area?

*Thank you for helpful comments, we appreciate your time and effort. We agree that our examination of the effect of prey distributions on predator occupancy may be limited by our ability to include all potential prey and that the effects of avian predators on sage-grouse demographics at our study site is unknown. However, because the removal of juniper at our study site was largely prompted by concerns over sage-grouse population conservation, we want to present our findings in the context of potential changes to the predator community for this species and highlight directions for future research. We added language in the discussion to this effect.*

*L 562.* *However, because we did not directly examine the impact of habitat-use by avian predators on sage-grouse demographics, future research on this topic will be an important next step for managers aiming to conserve populations of prey species.*

Detailed comments
Introduction
Overall, the introduction is very well written and does a great job of setting the study.
L 96-97 delete space before “For”

*Thank you, we corrected this formatting.*
You mentioned “For generalist predators that may utilize a wide variety of prey items, structural resources may be an important factor influencing habitat use.” But this also happens with forest-dwelling species and specialists. See Martínez-Hesterkamp S, Rebollo S, Pérez-Camacho L, García-Salgado G, Fernández-Pereira JM (2018) Assessing the ability of novel ecosystems to support animal wildlife through analysis of diurnal raptor territoriality.PLoSONE13(10):e0205799
Also, could you include a reference supporting those lines.

*Thank you, we adjusted the language of this sentence to be more general and added the following citation*

*L 96. “For avian predators that may utilize a wide variety of prey items, structural resources may be a primary factor influencing habitat use [22].”*

*Greenwald DN, Crocker-Bedford DC, Broberg L, Suckling KF, Tibbitts T. A review of northern goshawk habitat selection in the home range and implications for forest management in the western United States. Wildlife Society Bulletin. 2005;33: 120–148.*

In the title and abstract you mentioned the term generalist predators but in the introduction only referred to avian predators, except in L. 96-97. This may be confused for readers. I suggest being consistently with one term for predators. In the objectives L. 148-155 you use avian predators not generalist predators. I suggest the use of generalist predators in the manuscript.

*Thank you, we added language on L. 122 to indicate that when we refer to avian predators throughout the rest of the manuscript we are referring to our two generalist avian predator study species.*

*In recent years, increased abundance in sagebrush habitat of common ravens (Corvus corax) and red-tailed hawk (Buteo jamaicensis), two highly generalist avian predators (hereafter “avian predators”)*

Methods
Please include references in the section of Raven and raptors counts.

*Thank you, we added the following reference*

*L198. 46. Andersen, D.E. Survey techniques. In: Raptor research and management techniques. 2007. pp. 89-100.*

L 198 How much time did you spend in stationary counts? This was equal in all transects? Please describe.

*Thank you, we clarified these methods..*

*L. 195 10-minute observation periods*

L199 – 200. *Along each survey transect, the amount of time spent at stationary survey locations was consistent, but walking surveys between stationary survey locations varied based on terrain.*

L 215 Please mention the ground squirrel species

*Thank you, we moved our description of the ground squirrel species at our site from line 233 to line 216.*

L225-226 The transects for songbirds was an extension of 1.200 m, you mentioned that transects for avian predators had 800 m with 3 points counts within the transect in L 194-195. This it is not clear.

*Thank you, we clarified this sentence describing the placement of songbird survey stations along the avian predator survey transect:*

*L 227-229. Each survey transect for avian predators had three point counts stations placed at either end and in the middle of the survey transect.*

L 233-235 “Belding’s ground squirrels (Urocitellus beldingi), which occur in large semi-colonial populations, are the most common ground squirrel at our study site.” This could be used in the first paragraph of prey abundance. L. 214-216.

*See above*

Discussion
L 555-560 I suggest being more cautious with these lines, the relationship between the study and the presence of the sage-grouse is not clear.

L. 562-564 *Thank you, we added the following language about the limits for inference that our study provides.*

*“However, because we did not directly examine the impact of habitat-use by avian predators on sage-grouse demographics, future research on this topic will be an important next step for managers aiming to conserve populations of prey species.”*

7. PLOS authors have the option to publish the peer review history of their article ([what does this mean?](https://urldefense.com/v3/__https:/journals.plos.org/plosone/s/editorial-and-peer-review-process*loc-peer-review-history__;Iw!!JYXjzlvb!iLp3dbk4hWOvVv8rjAK9_MYyifNe-tzSHHYZWw0KGWMmBHhCZvFaorZsdLsA36EDITdELUVP2CrXrpWz$)). If published, this will include your full peer review and any attached files.


If you choose “no”, your identity will remain anonymous but your review may still be made public.


**Do you want your identity to be public for this peer review?** For information about this choice, including consent withdrawal, please see our [Privacy Policy](https://urldefense.com/v3/__https:/www.plos.org/privacy-policy__;!!JYXjzlvb!iLp3dbk4hWOvVv8rjAK9_MYyifNe-tzSHHYZWw0KGWMmBHhCZvFaorZsdLsA36EDITdELUVP2BOIkIEJ$).

Reviewer #2: No

Reviewer #3: No

[NOTE: If reviewer comments were submitted as an attachment file, they will be attached to this email and accessible via the submission site. Please log into your account, locate the manuscript record, and check for the action link "View Attachments". If this link does not appear, there are no attachment files.]

While revising your submission, please upload your figure files to the Preflight Analysis and Conversion Engine (PACE) digital diagnostic tool, [https://pacev2.apexcovantage.com/](https://urldefense.com/v3/__https:/pacev2.apexcovantage.com/__;!!JYXjzlvb!iLp3dbk4hWOvVv8rjAK9_MYyifNe-tzSHHYZWw0KGWMmBHhCZvFaorZsdLsA36EDITdELUVP2NBBPQqi$). PACE helps ensure that figures meet PLOS requirements. To use PACE, you must first register as a user. Registration is free. Then, login and navigate to the UPLOAD tab, where you will find detailed instructions on how to use the tool. If you encounter any issues or have any questions when using PACE, please email PLOS at [figures@plos.org](mailto:figures@plos.org). Please note that Supporting Information files do not need this step.
